# Microstructural Evolution and Tensile Properties of Al_0.3_CoCrFeNi High-Entropy Alloy Associated with B2 Precipitates

**DOI:** 10.3390/ma15031215

**Published:** 2022-02-06

**Authors:** Xiaodi Wang, Zhe Zhang, Zhengbin Wang, Xuechong Ren

**Affiliations:** 1 National Center for Materials Service Safety, University of Science and Technology Beijing, Beijing 100083, China; superaron@163.com; 2 CAS Key Laboratory of Nuclear Materials and Safety Assessment, Institute of Metal Research, Chinese Academy of Sciences, Shenyang 110016, China; zbwang12s@imr.ac.cn

**Keywords:** high-entropy alloy, microstructures, tensile properties, B2 precipitates, strengthening

## Abstract

The room-temperature strength of Al_0.3_CoCrFeNi high-entropy alloys (HEAs) is relatively low owing to its intrinsic fcc structure. In the present study, the as-cast HEAs were subjected to cold rolling and subsequent annealing treatment (800, 900, and 1000 °C) to adjust the microstructures and tensile properties. This treatment process resulted in the partial recrystallization, full recrystallization, and grain coarsening with increasing the annealing temperature. It was found that the large and spherical B2 precipitates were generated in the recrystallized grain boundaries of three annealing states, while the small and elongated B2 precipitates were aligned along the deformation twins in the non-recrystallized region of the 800 °C-annealing state. The former B2 precipitates assisted in refining the recrystallized grains to quasi ultra-fine grain and fine grain regimes (with the grain sizes of ~0.9, ~2.2, and ~7.2 μm). The tensile results indicated that the decreased annealing temperature induced the gradual strengthening of this alloy but also maintained the ductility at the high levels. The yield strength and ultimate tensile strength in 800 °C-annealed specimen were raised as high as ~870 and ~1060 MPa and the ductility was maintained at ~26%. The strengthening behavior derived from the heterogeneous microstructures consisting of quasi ultra-fine recrystallized grains, non-recrystallized grains, deformation twins, dislocations, and B2 precipitates. Current findings offer the guidance for designing the HEAs with good strength and ductility.

## 1. Introduction

The high-entropy alloys (HEAs) have attracted growing interest in research due to the novel alloy design concept in contrast to conventional alloys. This new class of alloys is composed of at least five principal elements with individual atomic concentrations ranging from 5% to 35% [1,2]. Due to the inherent composition complexity, the HEAs possess unique characteristics which are summarized as four core effects, i.e., high entropy effect, severe lattice distortion effect, sluggish diffusion effect, and cocktail effect. Such effects have been shown to greatly affect the microstructures and properties of HEAs [3,4]. The high entropy and reduced Gibbs free energy usually make HEAs form the simple solid solution structures (i.e., face-centered cubic (fcc), body-centered cubic (bcc), or hexagonal close-packed (hcp)) rather than intermetallic compounds. The fcc HEAs are also reported to show many excellent mechanical, physical, and chemical properties, which will promote their potential applications in various fields [5,6,7].

The Al_x_CoCrFeNi HEA is a widely investigated alloy system [8,9,10,11,12]. The alloys evolve from a single fcc phase to a single bcc phase with increasing the Al additions. Among them, Al_0.3_CoCrFeNi HEA is the single solid solution with fcc structure. It is reported that this HEA shows good mechanical properties such as high plasticity, work-hardening ability and cryogenic strength-ductility balance [10,11]. However, the strength of Al_0.3_CoCrFeNi HEA at room temperature is relatively low owing to the intrinsic characteristic of fcc structure. For example, its room-temperature yield strength is reported to be in the range of 150–350 MPa [12].

The thermo-mechanical processing (e.g., cold rolling and subsequent annealing) has been widely used to enhance the mechanical properties of Al_0.3_CoCrFeNi HEAs by tuning the microstructural evolution [13,14,15,16,17]. The microstructure characteristics are strongly dependent on the cold rolling reductions and annealing conditions. For instance, Annasamy et al. [13] designed a series of cold rolling (from 30% reduction to 70% reduction) and annealing (from 800 to 1050 °C) parameters for Al_0.3_CoCrFeNi HEAs and obtained the various microstructures including the recrystallization extents, grains, and precipitates. Such a richness of microstructures provides a larger chance to strengthen this alloy via adjusting these microstructures. Yasuda et al. [14] processed an ultrafine-grained microstructure with grain boundary B2 precipitates by 90% cold rolling and 800 °C annealing. This microstructure results in the excellent strength–ductility balance with tensile yield strength of ~800 MPa and uniform elongation of ~40% in Al_0.3_CoCrFeNi HEA. Gwalani et al. [15] obtained a more complicated microstructure with a hierarchical feature of ultra-fine grains, fine scale of precipitates (B2, bcc and *σ*) and nano-clusters through 90% cold rolling and 550 °C annealing. This contributes to a great improvement in the tensile yield strength from ~160 to ~1800 MPa.

It is noted above that the previous studies of strengthening fcc Al_0.3_CoCrFeNi HEAs were mainly focused on the fully recrystallized states accompanied by the grain refinement strengthening and precipitation strengthening. Recently, the partially recrystallized microstructures were reported to cause a good combination of strength and ductility in several fcc HEAs (such as CoCrNi, Al_0.1_CoCrFeNi, and CoCrFeMnNi) via thermo-mechanical treatment [18,19,20,21]. This is mainly caused by the significant back-stress effect produced during deformation. From this, we speculate that the present Al_0.3_CoCrFeNi HEAs with partial recrystallization should also possess the enhanced mechanical properties. To confirm it, the as-cast Al_0.3_CoCrFeNi HEAs were cold-rolled and annealed at elevated temperatures to obtain different microstructures including the partial and full recrystallization, and the corresponding tensile properties were investigated in this work.

According to the phase diagram [15], it is known that multiple types of precipitates (B2, L1_2_, *σ*, and bcc) are prone to form in the Al_0.3_CoCrFeNi HEA. It was reported that these precipitates played a key role in the recrystallization behaviors as well as the mechanical properties. To simplify the investigation, the annealing temperatures (800, 900, and 1000 °C) were selected in the dual-phase region of fcc phases and B2 phases. Three annealing temperatures led to the partial recrystallization, full recrystallization, and grain coarsening accompanied by B2 precipitation. The microstructural characteristics including the grain size, size and percentage of B2 phases, etc. were measured in three annealing states by microscopic analyses. The strength and ductility were measured and compared based on the tensile results. Finally, the relationship between the microstructures and tensile properties was established in this HEA.

## 2. Experimental Procedure

### 2.1. Materials and Sample Preparation

The magnetic levitation induction technique was utilized to melt a mixture of elemental Al, Co, Cr, Fe, and Ni with the purity of >99.9% in a high-purity argon atmosphere, to produce the Al_0.3_CoCrFeNi (at.%) HEA ingots. The melting temperature was 1600 °C. The ingots were re-melted four times to ensure the chemical homogeneity and then poured into the rectangular steel mold at room temperature. The pouring temperature was 1400 °C. It is noted that the venting plug was used in the mold to vent the air, for ensuring the quality of HEA plates. The fabricated HEA plates had the dimensions of 150 mm (length) × 60 mm (width) × 20 mm (thickness). The plates were homogenized at 1150 °C for 24 h followed by water cooling, and then cold-rolled to ~70% thickness reduction along the longitudinal direction. Then the cold-rolled sheets were annealed at various temperatures of 800, 900, and 1000 °C for 60 min followed by water cooling, respectively, to generate different microstructures.

The dog-bone-shape plate tensile specimens with a gauge length of 15 mm, a width of 3.5 mm, and a thickness of 3.7 mm were cut by electric discharging machine. The four surfaces of specimens were ground down to 1500-grit SiC abrasive paper before tensile testing. The electron backscattering diffraction (EBSD) specimens were fabricated by mechanical grinding and subsequent vibration polishing (ATM SAPHIR Vibro, Germany) using colloidal silica suspension with a particle size of 60 nm. The specimens for transmission electron microscopy (TEM) observation with the original dimensions of 5 × 5 × 0.5 mm^3^ were mechanically ground to the thickness below ~50 μm and then ion-milled using a Gatan 691 equipment.

### 2.2. Microstructural Characterization and Mechanical Testing

The crystal structures of as-cast and annealing specimens were characterized by X-ray diffraction (XRD, Rigaku smartlab diffractometer, Tokyo, Japan) using Cu Kα radiation in the 2θ range of 20°–100° at a scanning speed of 4°/min. The initial microstructures were analyzed by scanning electron microscopy (SEM, ZEISS Merlin compact, Oberkochen, Germany) equipped with an EBSD detector and a TEM (FEI Tecnai G2 F20, Hillsboro, Oregon State, America) equipped with an X-ray energy dispersive spectrometer (EDS). The step size for EBSD measurement was 0.1 μm and the corresponding data were analyzed by Channel5 analysis software.

The uniaxial tensile tests were carried out by an MTS (Shenzhen, China) E44 testing machine at room temperature. The extensometer was attached to gauge section to track the tensile strain. All the tensile tests were performed at the strain rate of 10^−3^/s and repeated three times to ensure the data reliability. The hardness values were measured using a Vickers hardness tester with a load of 200 g and a dwell time of 15 s. The fifteen points were selected for each specimen state in the hardness test, to ensure the result accuracy.

## 3. Results

### 3.1. Microstructures

**Figure 1:**
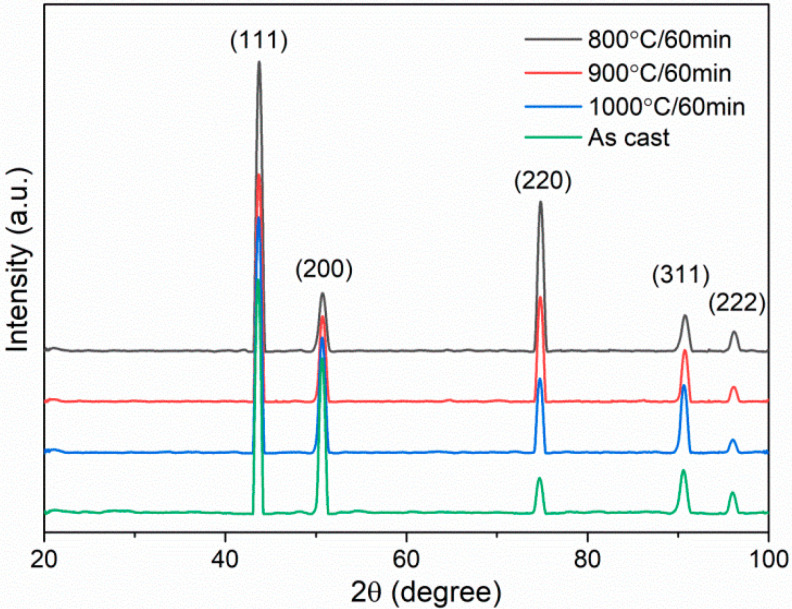
XRD curves of as-cast HEA specimens and specimens after cold rolling and subsequent annealing.

Figure 1 shows the XRD curves of different HEA specimen states. The Bragg diffraction peak positions of specimens after cold rolling and subsequent annealing treatment are similar to those in the as-cast specimen, both demonstrating the fcc single-phase solid solution structure (referring to PDF33-0397). No peaks representing other phases are found. It seems that the precipitates are not generated after cold rolling and annealing treatment. More characterization methods will be used to identify the microstructures in the following sections.

**Figure 2:**
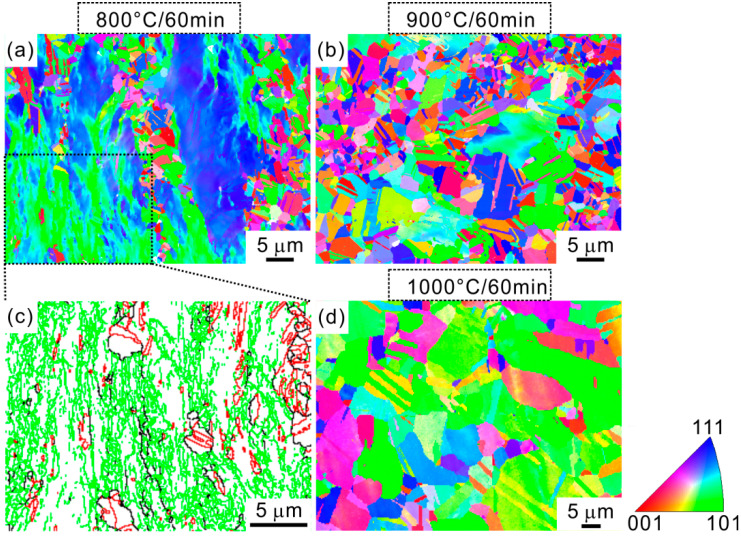
Inverse pole figure (IPF) maps recorded from (**a**) 800 °C, (**b**) 900 °C, and (**d**) 1000 °C annealed HEA specimens. (**c**) is grain boundary map from rectangle region in (**a**).

Figure 2 reveals the microstructural evolution with increasing the annealing temperature through EBSD measurement. The inverse pole figure (IPF) map of 800 °C-annealed specimen (Figure 2a) exhibits two different regions, i.e., one is the extended region and the other is the region consisting of fine recrystallized grains. Figure 2c is the grain boundary map from the extended region in Figure 2a. The green, black, and red lines represent the low-angle grain boundaries (LAGBs), high-angle grain boundaries (HAGBs), and twin boundaries (TBs), respectively. It is clear that the LAGBs occupy the dominant proportion, which suggests that the extended region is non-recrystallized region. This means that the 800 °C-annealing treatment cannot cause the complete recovery and recrystallization in the cold-rolled specimen, eventually producing the partially recrystallized microstructure. As the annealing temperature is raised to 900 °C, the specimen is fully recrystallized in the form of randomly oriented equiaxial grains. With further increasing the annealing temperature to 1000 °C, the recrystallized grains are coarsened. It is concluded that the recrystallization is strongly dependent on the annealing temperatures [20], and the microstructures of present HEA transform from the partial recrystallization to full recrystallization with increasing the annealing temperature.

**Figure 3:**
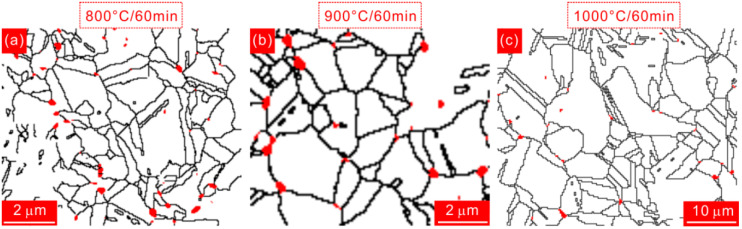
Phase maps of HEA specimens annealed at (**a**) 800 °C, (**b**) 900 °C, and (**c**) 1000 °C. The precipitate phases are indicated by red colors. The lengths of scale bars in (**a**–**c**) are identical.

**Figure 4:**
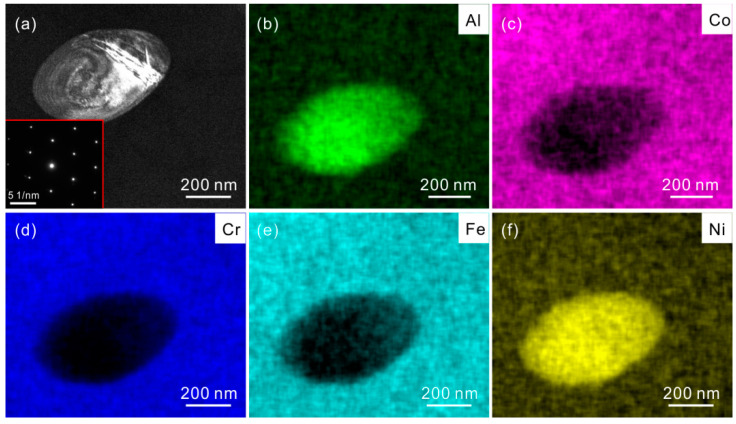
TEM-EDS elemental mapping for precipitate phase. (**a**) TEM image of precipitate and the inset is corresponding selected area electron diffraction (SAED) pattern; (**b**) Al, (**c**) Co, (**d**) Cr, (**e**) Fe and (**f**) Ni.

The phase maps of different HEA states, which are analyzed from EBSD data, are shown in Figure 3. Figure 3a is selected from the recrystallized region in 800 °C-annealed specimen. This specimen is composed of matrix phase (white region) and precipitates with spherical shape (red region). A large amount of precipitates are decorated along the grain boundaries. The precipitate distributions in 900 °C- and 1000 °C-annealed specimens are similar to that in 800 °C-annealed specimen, as shown in Figure 3b,c. The precipitate size seems to increase with increasing the annealing temperature. The detailed values of size and volume fraction of precipitates will be measured and compared in the following sections. Figure 4 reveals the elemental mapping for this precipitate through TEM–EDS method. It is seen that the concentrations of Al and Ni in the precipitate region are obviously higher than those in the surrounding region. The precipitate is enriched with Al–Ni segregation. This precipitate is further identified to be bcc B2 phase via selected area electron diffraction (SAED) pattern in the inset of Figure 4a. So it is basically determined that the precipitates are the AlNi-type B2 phase. It is reported that Al atom is easy to precipitate from fcc matrix phase due to its larger atomic radius than other elements in Al_0.3_CoCrFeNi system. Besides, owing to the high negative enthalpy of mixing between Al and Ni, Al and Ni atoms are prone to be combined, finally forming the AlNi-type B2 phase [11,22,23].

**Figure 5:**
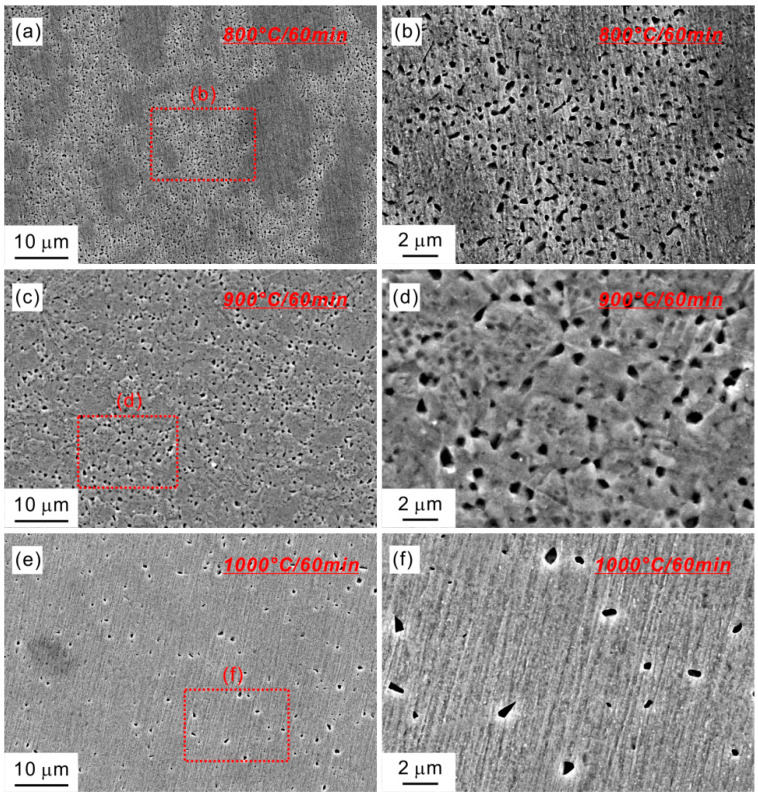
SEM micrographs of HEA specimens annealed at (**a**) 800 °C, (**c**) 900 °C, and (**e**) 1000 °C. (**b**,**d**,**f**) are magnified images in boxed regions of (**a**,**c**,**e**).

The step size is set to be low in the EBSD measurement, for examining the fine precipitates. It is not cost-effective to measure the EBSD data in a large specimen area using the low step size. So we further investigated the microstructural evolution in various annealing temperatures by observing the SEM micrograph, as shown in Figure 5. Obviously, two different regions exist in a large SEM view field after 800 °C annealing. This is consistent with above EBSD results, further demonstrating the partially recrystallized microstructure. One region is full of spherical B2 precipitates (Figure 5b), which is identified as recrystallized region according to the phase map (Figure 3a). Different from the 800 °C-annealed specimen, the B2 precipitates are uniformly distributed in the 900 °C- and 1000 °C-annealed specimens, as shown in Figure 5c–f. This is related to their completely recrystallized microstructures. It is also seen that the volume fraction of B2 precipitates is reduced with increasing the annealing temperature from 900 to 1000 °C.

**Figure 6:**
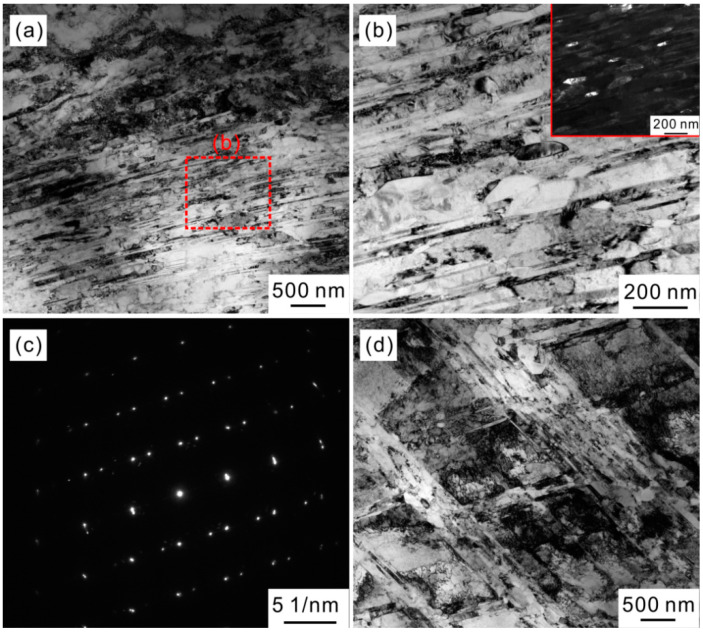
(**a**,**d**) TEM images of microstructures in non-recrystallized region of 800 °C-annealed specimen, showing the deformation twins, dislocations and precipitates; (**b**) magnified image of red rectangle region in (**a**); (**c**) corresponding selected area electron diffraction (SAED) pattern taken from the twin in (**a**); The inset is the dark field micrograph of (**b**).

In fact, there exists a weak contrast change in the non-recrystallized region of 800 °C-annealed specimen (Figure 5b). To further examine the structure information in this region, the corresponding TEM observation is conducted, as shown in Figure 6. Figure 6a reveals the microstructure of non-recrystallized region. There are a high density of dislocations and parallel deformation twins in this region. These should be the microdefects induced by cold rolling, which are not sufficiently annihilated during the recovery and recrystallization process [18,24,25,26]. Some elongated B2 precipitates with the size smaller than grain-boundary precipitates are aligned along the deformation twins, as shown in Figure 6b and its inset. In addition, this type of B2 precipitates also tend to form in the cross points of intersected twins (Figure 6d). Combined with above results, the B2 precipitates in the partially recrystallized microstructure are divided into two types, namely, the large and spherical B2 along the grain boundaries in the recrystallized region and the small and elongated B2 along the deformation twins in the non-recrystallized region. The similar features of B2 precipitates were also reported in the previous studies about the non-recrystallized microstructures of HEAs [24,25,26]. Since the nucleation barrier is high owing to the large fcc/B2 interface energy, the B2 phases tend to precipitate in above inhomogeneous locations.

**Figure 7:**
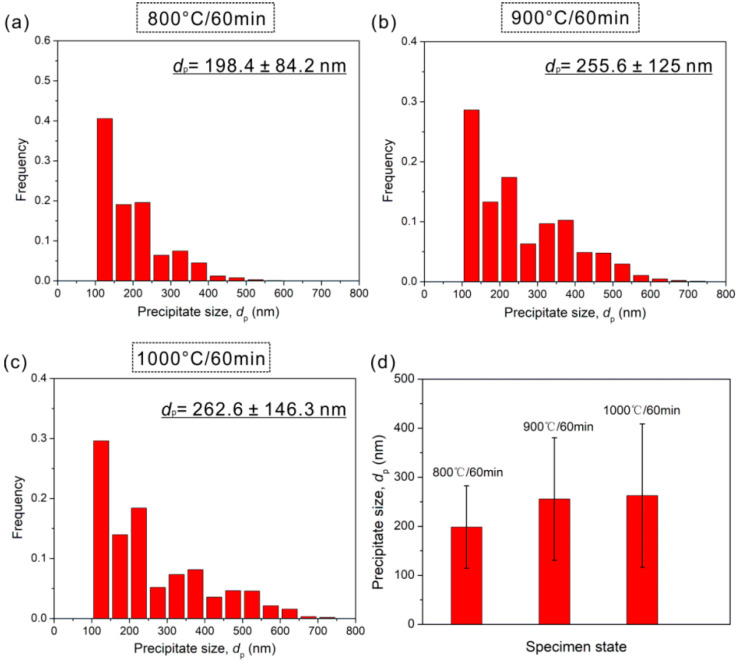
The histograms of precipitate size distribution in HEAs annealed at (**a**) 800 °C, (**b**) 900 °C, and (**c**) 1000 °C; (**d**) precipitate size as a function of annealing temperature.

**Figure 8:**
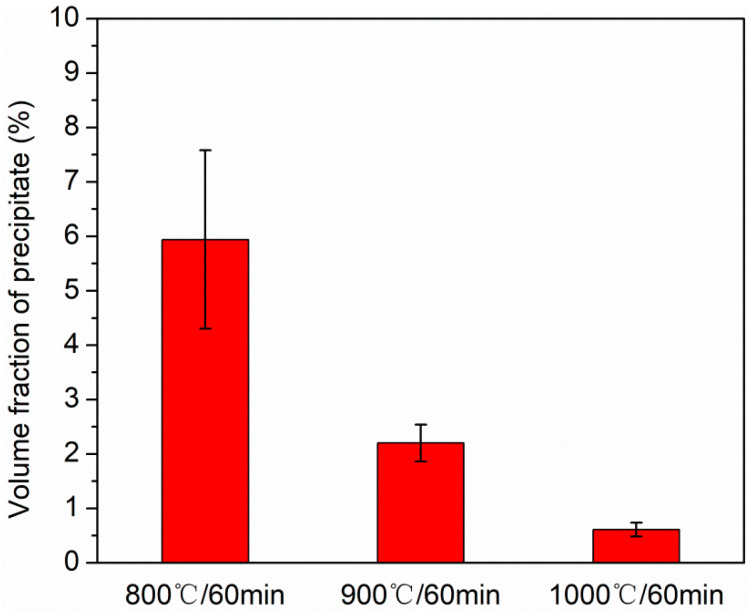
Volume fraction of precipitate in different HEA states.

Based on above results, it is known that the B2 precipitates are the key microstructures in cold-rolled and annealed specimens. In order to precisely determine the size and volume fraction of B2 precipitates, we count four large SEM view fields of 120 × 80 μm^2^ via image J software in the recrystallized region of 800 °C-annealed specimen, 900 °C-annealed specimen and 1000 °C-annealed specimen, respectively. The results for different states are listed in Figure 7. The precipitates sizes in term of equivalent diameter, *d_p_* are ~198 ± 84, ~256 ± 125, and ~263 ± 146 nm for 800 °C-, 900 °C-, and 1000 °C-annealing, respectively. It is seen that the value of *d_p_* increases by ~33% when varying the annealing temperature from 800 to 1000 °C. Figure 8 reveals the volume fractions of precipitates in different states. The volume fractions of 800 °C-, 900 °C-, and 1000 °C-annealed specimens are ~5.9 ± 1.6%, ~2.2 ± 0.3%, and ~0.6 ± 0.1%. Obviously, the volume fraction exhibits a decreasing trend with increasing the annealing temperature. Here, we also know that the small size and amounts probably lower than XRD resolution may be the reason why the XRD fails to detect the precipitates (Figure 1) [17,27]. In addition, more studies about the kinetics of B2 precipitation and dissolution should be conducted to understand well the variation trend of the size and volume fraction with increasing the annealing temperature.

**Figure 9:**
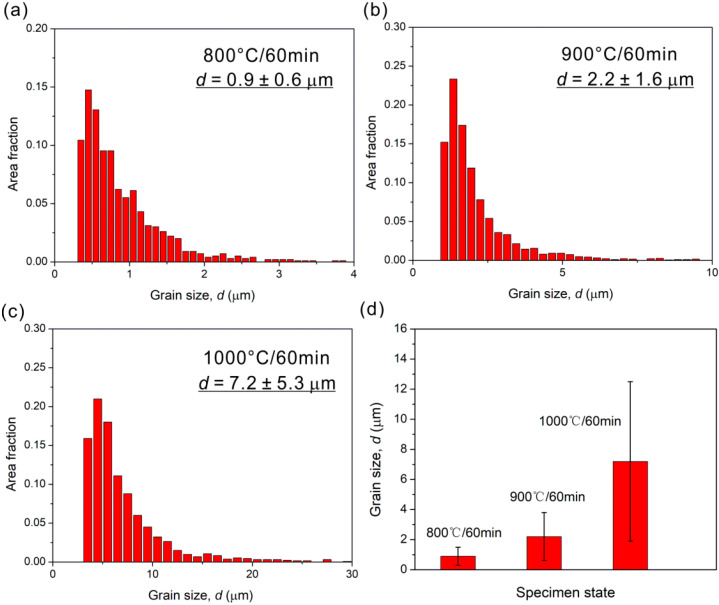
The histograms of grain size distribution in HEAs annealed at (**a**) 800 °C, (**b**) 900 °C, and (**c**) 1000 °C; (**d**) grain size as a function of annealing temperature.

The grain sizes of three HEA states are obtained through EBSD analysis, as shown in Figure 9. The grain size, *d* in the recrystallized region of 800 °C-annealed specimen is ~0.9 ± 0.6 μm; and the values of *d* in 900 °C- and 1000 °C-annealed specimens are ~2.2 ± 1.6 and ~7.2 ± 5.3 μm, respectively. The grain sizes show an increasing trend with increasing the annealing temperature. In addition, the 800 °C-annealed specimen is in the quasi ultra-fine grain regime and the other states are in the fine grain regime. The grains are greatly refined by the cold rolling and subsequent annealing treatment, relative to the as-cast specimen [12].

**Figure 10:**
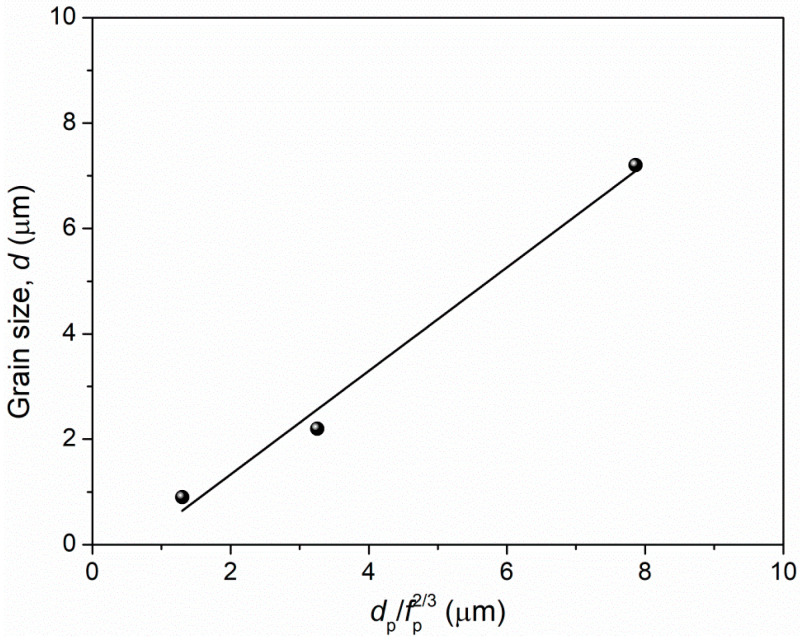
Relation between grain size and precipitate characteristics.

The precipitates along the grain boundaries play a key role in suppressing the grain growth during the recrystallization process, which may be the reason for the fine-scale grains in three states. It is previously reported that the grain size, *d*, is proportional to $\frac{d_{p}}{f_{p}^{2/3}}$ [14,28], where *d_p_* and *f_p_* are the size and volume fraction of precipitates. Yasuda et al. [14] designed a series of grain sizes from ultra-fine grain regime to fine grain regime to coarse grain regime through tuning the values of *d_p_* and *f_p_*. Here we also plot the relationship between the *d* vs. $\frac{d_{p}}{f_{p}^{2/3}}$ in Figure 10, and confirm this proportional relationship in present alloy.

### 3.2. Mechanical Properties

**Figure 11:**
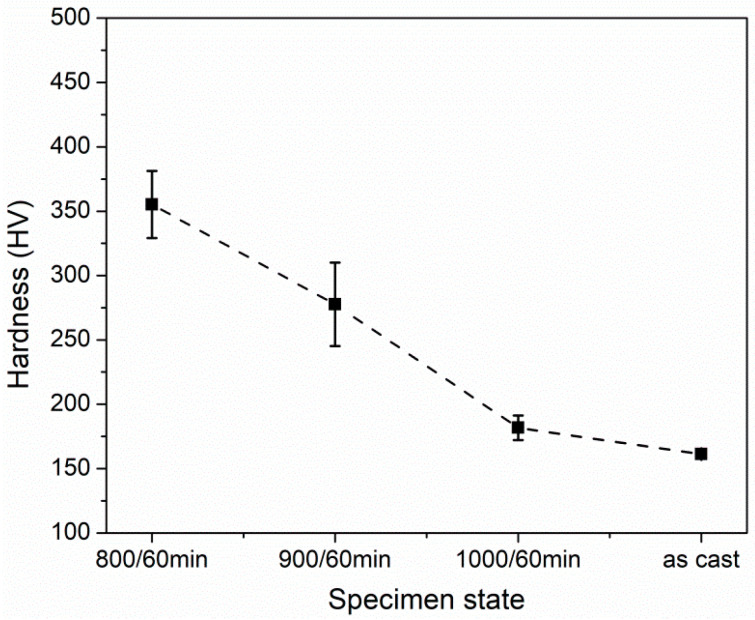
Vicker hardness variation in as-cast and different annealed HEA states.

**Figure 12:**
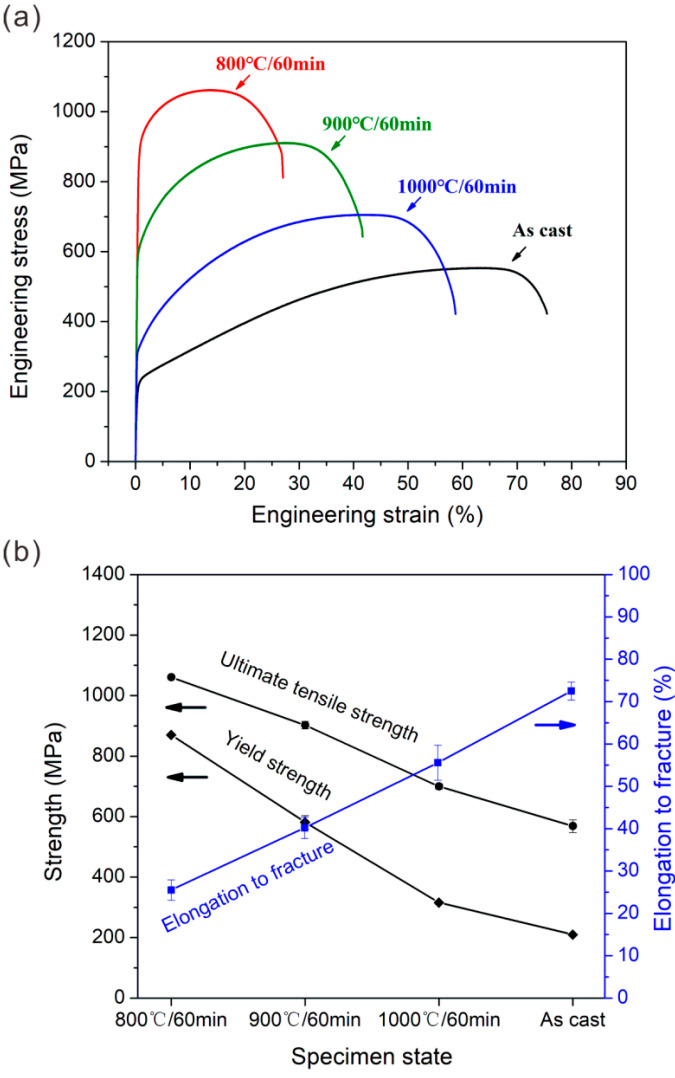
(**a**) Engineering tensile stress-strain curves and (**b**) yield strength, ultimate tensile strength and elongation to fracture in as-cast and different annealed HEA states.

Figure 11 shows the hardnesses in different HEA states. The specimens after cold rolling and annealing possess the higher hardnesses, as compared with the as-cast state. And as the annealing temperature decreases, the hardness gradually increases. To further evaluate their mechanical properties, we conduct the uniaxial tensile tests and obtain the engineering stress–strain curves, as shown in Figure 12a. For a clear cognition, the yield strength, ultimate tensile strength, and elongation to fracture in different states are also measured and plotted in Figure 12b. The cold rolling and subsequent annealing leads to the significant enhancement in the yield strength and ultimate tensile strength, relative to the as-cast state. This strength variation trend is well consistent with the hardness result. In addition, the specimens after cold rolling and annealing still maintain the large tensile ductility. The yield strength and ultimate tensile strength can be raised to ~870 ± 1.6 and ~1060 ± 2.4 MPa in 800 °C-annealed specimen, respectively, which are four times and twice as high as those in as-cast specimen, ~210 ± 4.3 and ~569 ± 21.1 MPa. And the 800 °C-annealed specimen also owns the large ductility of ~25.5 ± 2.4%. This result indicates that a good combination of strength–ductility can be achieved in the partially recrystallized microstructure of present alloy.

**Figure 13:**
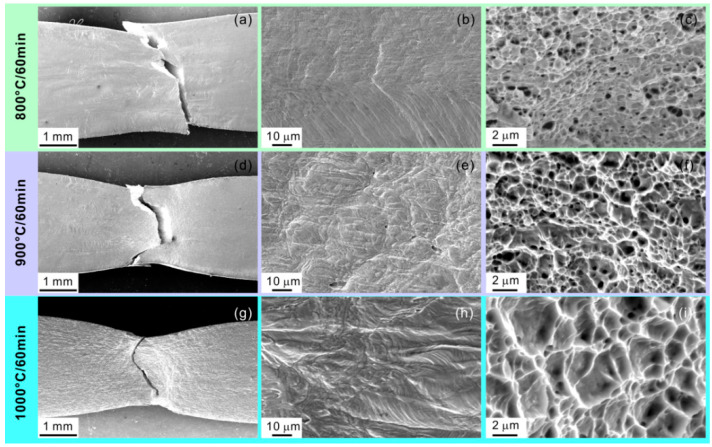
SEM images of surface damage and fracture surface features in (**a**–**c**) 800 °C-, (**d**–**f**) 900 °C-, and (**g**–**i**) 1000 °C-annealed HEA specimens.

Figure 13 shows the comparison of surface damage and fracture surface features in different HEA states, giving a further supplement of tensile results. It is seen that the necking and shear fracture occur in 800 °C-annealed specimen (Figure 13a). The specimen surface exhibits the extensive slipping traces in the non-recrystallized and recrystallized regions (Figure 13b). For its fractography, there are a large amount of shallow and small dimples (Figure 13c), which reflect the ductile fracture. Above features indicate the good plastic deformation ability of 800 °C-annealed specimens before fracture, well accounting for the ductility of ~25.5%. The 900 °C- and 1000 °C-annealed specimens exhibit the larger necking with the severer slipping on the specimen surface and the larger dimples on the fracture surface (Figure 13d–i). This feature variation indicates the better plastic deformation ability with increasing the annealing temperature, which agrees well with the tensile plasticity.

## 4. Discussion

### 4.1. Microstructure Analysis with Increasing Annealing Temperature

Based on above multiple microstructural characterizations using the XRD, EBSD, SEM, and TEM, it is determined that the present HEA specimens are subjected to partial recrystallization, full recrystallization, and grain coarsening accompanied by the variation of structural characteristics of B2 precipitates, with increasing the annealing temperature from 800 to 1000 °C. With further increasing the annealing temperature to 1150 °C, the grains continue to grow to coarse-grain scale (~83.1 μm) but the precipitates vanish, as reported in our previous studies [29]. In the following, we attempt to elaborate the microstructural evolution process of present alloy, by combining the present findings in this work and previous results in other references [13,14,24,29,30].

The microstructural evolution strongly depends on the recrystallization kinetics and precipitation kinetics [13,30]. The cold-rolled specimen is partially recrystallized with B2 precipitation after 800 °C annealing for 60 min. This means that the annealing temperature and time reach the critical condition for the recrystallization onset and B2 phase precipitation. The B2 phases tend to precipitate along the grain boundaries in the recrystallized region and along the deformation twins in the non-recrystallized region. The grain-boundary B2 phases suppress the grain growth, finally resulting in the quasi ultra-fine grains in the recrystallized region. The large distortion energy induced by cold rolling as well as the fast elemental diffusion channel induced by extensive dislocations and deformation twins promotes the formation of B2 phases in the non-recrystallized region [20,26]. The formation of these B2 phases will consume a lot of energy, which plays a role in retarding the recrystallization. Annasamy et al. [13] calculated and compared the recrystallization activation energy of Al_0.3_CoCrFeNi HEAs with and without B2 phases. They found that the recrystallization activation energy was obviously higher in the HEAs with B2 phases. Moreover, the recrystallization activation energy in this HEA with B2 phases also exceeds other typical conventional alloys. Thus, the formation of B2 phases increases the difficulties for recrystallization.

When the annealing temperature is raised to 900 °C, the temperature reaches the critical condition for full recrystallization and is also above the critical condition for B2 phase precipitation. For this annealing temperature, the full recrystallization finishes accompanied with the precipitation of a large amount of B2 phases along the grain boundaries. These phases provide the strong pining effect on the movement of the boundaries [13,14], leading to the formation of small grains with fine-grain scale. When the annealing temperature reaches 1000 °C, this temperature may be above the critical condition for full recrystallization and close to the critical condition for B2 dissolution. The volume fraction of B2 phases is apparently low (~0.6 ± 0.1%). This weakens the pinning effect of B2 phases on grain boundary migration, finally leading to the grain coarsening. As the annealing temperature is increased to 1150 °C, the grains further grow and the B2 phases disappear [29]. This means that this annealing temperature probably reaches or exceeds the critical condition for B2 dissolution. Above qualitative analyses are mainly based on the present and previous results, and more detailed investigations should be done to determine the critical conditions for recrystallization onset and completion as well as B2 precipitation and dissolution in Al_0.3_CoCrFeNi HEAs in future. It is believed that it is of great importance for quantitatively tuning their microstructures.

### 4.2. Strengthening Mechanism in 900 °C- and 1000 °C-Annealed Specimens

One typical feature for Al_0.3_CoCrFeNi HEA is that it encompasses a remarkably large number of phase fields (e.g., fcc, B2, L1_2_, bcc and *σ* phases) as a function of temperature, according to the phase diagram [15]. It means that this alloy composition is prone to decompose to more than one phase after suitable annealing treatment. This gives a large chance to improve the strength via tuning the precipitate characteristics, owing to the fact that the precipitates can act as the obstacles for dislocation motion. Indeed, several studies have reported the enhanced strength in this alloy by taking advantage of the precipitation strengthening effect [15,16,31]. Nevertheless, a recent study shows that the effect of precipitates on the strength is not obvious in the alloy with same composition [14]. The researchers obtained various Al_0.3_CoCrFeNi HEA specimens with different grain sizes through controlling the precipitate formation and plotted the Hall–Petch curve by using the yield strength–grain size data. This curve is straight regardless of the presence or absence of precipitates, which indicates that the precipitation strengthening may be small or neglected. In the present work, the 900 °C- and 1000 °C-annealed specimens exhibit the fine recrystallized grains with B2 precipitates along the grain boundaries. Their possible strengthening mechanisms are the grain boundary strengthening and precipitation strengthening. Considering that the precipitation strengthening effect is obscure in this alloy, as mentioned above, the contributions of grain boundary strengthening and precipitation strengthening to the strength should be further explored.

**Figure 14:**
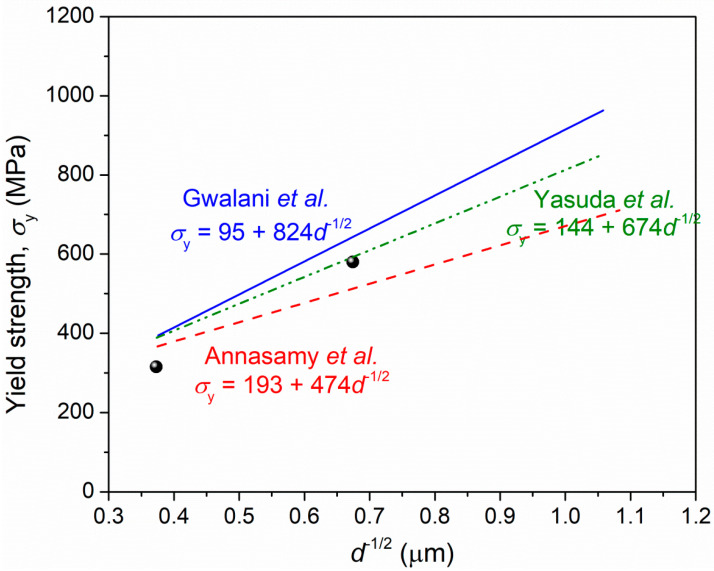
Hall–Petch relationship plotted based on previous references [13,14,16] and the present work data.

The Hall–Petch relationship is suitable for describing the grain boundary strengthening. Since the present data are not sufficient to plot the Hall–Petch curve, we collect the related results of Al_0.3_CoCrFeNi HEAs in the previous references as possible as we can [13,14,16], as shown in Figure 14. It is seen that the values of lattice friction coefficient (curve intercept) and H-P coefficient (curve slope) are varying in different references. The reason for the coefficient difference is complicated, including the material quality, data dispersion, etc. Moreover, the H-P coefficient, *k_H-P_* is calculated through the following equation, which is described as [19]:

(1) $$k_{H-P}=3M\sqrt{\frac{\pi m_{s}Gb\tau_{c}\left(2-\upsilon \right)}{4\left(1-\upsilon \right)}},$$

where M = 3.06 is the Taylor factor [19], *m_s_* =2.238 is a constant for fcc crystals [19], *G* = 80 GPa is the shear modulus [15], *b* = 2.55 × 10^−10^ m is the Burgers vector [15], *τ_c_* = 80 MPa is the critical resolved shear stress for slip [32], and *υ* = 0.3 is Poisson’s ratio [33]. Substituting these values into Equation (1), the value of *k_H-P_* is calculated to be ~766 MPa·μm^1/2^, which is between the reported values of ~824 MPa·μm^1/2^ and ~474 MPa·μm^1/2^ in the references.

The data of yield strength vs. grain size in 900 °C- and 1000 °C-annealed specimens are added in Figure 14. It is seen that one data point, as indicated by the black sphere, is localized in the region between the reported Hall–Petch curves; the other data point is slightly lower than this region. This suggests that the grain boundary strengthening mechanism is mainly responsible for the enhancement of yield strength in 900 °C- and 1000 °C-annealed specimens. In other words, the precipitate strengthening plays a weak or neglected role in strengthening the present alloy.

In order to further confirm this conclusion, the contribution from precipitation strengthening to yield strength is calculated in the following. It is known that the precipitation strengthening is generally divided into the particle shearing mechanism and the Orowan bypassing mechanism, which depend on the prevention modes of precipitates in the dislocation motion [31,34]. The particle shearing mechanism occurs in the condition that the precipitate particles are small and coherent with the matrix, while the Orowan bypassing mechanism occurs in other conditions. As mentioned above, the B2 precipitates possess a bcc structure and the matrix phases have an fcc structure. The B2 precipitates and matrix phases were reported to follow the Kurdjumov–Sachs (K–S) orientation relationship in present HEA, i.e., the {111}fcc // {110}B2 and <110>fcc // <111>B2 [12,23,24]. The B2 precipitates are in the submicron scale (~250 nm) and share the semi-coherent interface with fcc matrix. So the precipitation strengthening should be dominated by the Orowan bypassing mechanism. These precipitates can well inhibit the dislocation slipping. The contribution of precipitation strengthening from Orowan bypassing mechanism, *σ_p_* can be expressed as follows [35]:

(2) $$\sigma_{p}=M\frac{0.4Gb}{\pi \sqrt{1-\upsilon }}\frac{\ln\left(\frac{2\sqrt{\frac{2}{3}}r}{b}\right)}{\lambda_{p}},$$

(3) $$\lambda_{p}=2\sqrt{\frac{2}{3}}r\left(\sqrt{\frac{\pi }{4f}}-1\right),$$

where M = 3.06 is the Taylor factor [19], *G* = 80 GPa is the shear modulus [15], *b* = 2.55 × 10^−10^ m is the Burgers vector [15], *υ* = 0.3 is Poisson’s ratio [33], *r* is the radius of precipitates, *f* is volume fraction of precipitates, and *λ_p_* is the inter-precipitate spacing. Based on the above equation, the values of *σ_p_* are calculated to be ~61.2 and ~28.8 MPa for 900 °C- and 1000 °C-annealed specimens, which occupy ~10.5% and ~9.1% of their yield strength, respectively. Obviously, the contribution of precipitation strengthening to yield strength is very low, which is consistent with above analyses. In fact, the contribution levels of precipitation strengthening are greatly associated with the type, size and volume fraction of precipitates [36,37]. How to optimize these precipitate parameters is the key to further enhance the strength.

### 4.3. Strengthening Mechanism in 800 °C-Annealed Specimen

Different from the fully recrystallized microstructures in 900 °C- and 1000 °C-annealed specimens, the 800 °C-annealed specimen exhibits partially recrystallized microstructure which consists of the recrystallized region and non-recrystallized region. The partially recrystallized material can be regarded as a “composite” material [28,35]. The strengthening formula based on the rule of mixtures can be simply expressed as:

(4) $$\sigma_{y}=\sigma_{yn}f_{n}+\sigma_{yr}f_{r},$$

where *f_n_* and *f_r_* are the volume fractions of non-recrystallized and recrystallized regions, respectively, and *σ_yn_* and *σ_yr_* represent the yield strengths of non-recrystallized and recrystallized regions, respectively.

The non-recrystallized region consists of a high density of dislocations, deformation twins and B2 precipitates (Figure 6). These residual dislocations will hinder the movement of new dislocations, leading to the dislocation strengthening, *σ_dn_*. Similar to the role of grain boundaries, the deformation twins act as the obstacle to dislocation movement, leading to the Hall–Petch strengthening, *σ_gn_*. In addition, the B2 precipitates can produce the precipitation strengthening, *σ_pn_* as mentioned above. It is reported that the yield strength of materials can be estimated by a simple sum of different strengthening contributions from the individual crystal defects [38,39]. So, the contributions of *σ_dn_*, *σ_gn_*_,_ and *σ_pn_* to the yield strength in the non-recrystallized region, *σ_yn_* can be linearly summed up as follows:

(5) $$\sigma_{yn}=\sigma_{dn}+\sigma_{gn}+\sigma_{pn}.$$

On the other hand, the recrystallized region consists of quasi ultra-fine grains and B2 precipitates. The grain boundary strengthening, *σ_gr_* and precipitation strengthening, *σ_pr_* are responsible for the strengthening in the recrystallized region. So the *σ_yr_* can be expressed as:

(6) $$\sigma_{yr}=\sigma_{gr}+\sigma_{pr}.$$

Substituting Equations (5) and (6) into Equation (4), the yield strength, *σ_y_* can be expressed as:

(7) $$\sigma_{y}=\left(\sigma_{dn}+\sigma_{gn}+\sigma_{pn}\right)f_{n}+(\sigma_{gr}+\sigma_{pr})f_{r}.$$

It is seen from Equation (7) that multiple strengthening mechanisms result in the enhanced strength in 800 °C-annealed specimen. This intrinsically derives from the heterogeneous microstructures, which are composed of the quasi ultra-fine grains, large non-recrystallized grains, dense dislocations/deformation twins, and B2 precipitates with different shapes and sizes. In addition, the heterogeneous microstructure can also produce the significant back stress during deformation, which leads to the strength enhancement [19,20,26]. The back-stress strengthening may be another important supplement for the strengthening in 800 °C-annealed specimen.

## 5. Conclusions

In this study, the microstructures and mechanical properties of Al_0.3_CoCrFeNi HEAs after cold rolling and subsequent annealing at 800, 900, and 1000 °C were systematically investigated. The detailed conclusions were draw as follows.

(1) The microstructures in 800 °C-, 900 °C-, and 1000 °C-annealed specimens showed the partial recrystallization, full recrystallization, and grain coarsening, respectively.

(2) Two types of B2 phases were precipitated after cold rolling and subsequent annealing. The sizes of B2 precipitates along the grain boundaries increased but their volume fractions decreased with increasing the annealing temperature. These grain-boundary precipitates refined the grain sizes into the quasi ultra-fine grain regime (~0.9 μm) and fine grain regime (~2.2 and ~7.2 μm) in 800 °C-, 900 °C-, and 1000 °C-annealed specimens.

(3) The 900 °C- and 1000 °C-annealed specimens with the recrystallized grains and B2 precipitates possessed the higher strength than the as-cast one. The precipitation strengthening contributions were calculated to occupy ~10.5% and ~9.1% of yield strengths, respectively. It seems that the precipitation strengthening plays a weak role in enhancing the strength, relative to grain boundary strengthening in two annealing states.

(4) The 800 °C-annealed specimens with partially recrystallized microstructure showed the yield strength of ~870 MPa and ultimate tensile strength of ~1060 MPa as well as the ductility of ~26%. The superior strength was attributed to the heterogeneous microstructures, which are composed of the quasi ultra-fine recrystallized grains decorated with spherical B2 precipitates along the grain boundaries and non-recrystallized grains decorated with deformation twins, dislocations and elongated B2 precipitates along the deformation twins.

## Data Availability

Not applicable.
